# Prevalence of dementia among older persons in Northern Uganda, a cross-sectional study

**DOI:** 10.4314/ahs.v26i1.10

**Published:** 2026-03

**Authors:** Dan Langoya Oriba, Matovu Stephen Mwonge, Robert Mukisa, Martin N Kaddu-Mukasa, Felix Bongomin, Abdu K Musubire, Mark Kaddumukasa

**Affiliations:** 1 Department of Internal Medicine, School of Medicine, Makerere University College of Health Sciences, Kampala, Uganda; 2 Department of Internal Medicine, St. Mary's Hospital Lacor, Gulu, Uganda; 3 Division of Neurology, Directorate of Internal Medicine, Mulago Specialized National Referral and Teaching Hospital, Kampala, Uganda; 4 Division of Neurology, Directorate of Internal Medicine, Kiruddu National referral Hospital, Kampala, Uganda; 5 Department of Medical Microbiology & Immunology, Faculty of Medicine, Gulu University, Gulu, Uganda

**Keywords:** Dementia, Sub-Saharan Africa, Northern Uganda

## Abstract

**Background:**

The burden and clinical correlates of dementia are unknown in Uganda. We therefore determined the prevalence and factors associated with dementia among older persons in Northern Uganda.

**Methods:**

In a cross-sectional study, adults aged 60 years and above attending the medical outpatient clinic at a tertiary Hospital from March 2022 to April 2022 were enrolled. The prevalence of dementia was determined using the Intervention for Dementia in Elderly Africans (IDEA) and the Instrumental Activities of Daily Living (IADL) tool henceforth presented as IDEA-IADL. Factors of association were collected using a standardized questionnaire.

**Results:**

A total of 271 eligible participants were enrolled into the study. Majority were age 60-69 years (153/271) and of male gender (165/271). The prevalence of dementia was 17.7% (48/271), severe dementia 7.4%, (20/271), moderate dementia 10.3%, (28/271). Factors independently associated with dementia were: participants aged between 80-89 years; (adjusted odds ratio (aOR): 9.7; 95% Confidence Interval (CI): 4.08-23.07, p< 0.001), family history of dementia (aOR: 3.4, 95CI: 1.62-7.04, p=0.001). history of depression (aOR: 2.7, 95CI: 1.40-5.02, p=0.003). Physical activity and cognitive exercise were associated protective factors with (aOR 0.1, 95CI: 0.03-0.24, p< 0.001) and (aOR 0.2, 95CI: 0.06-0.53, p<0.002) respectively.

**Conclusion:**

There burden of dementia among older persons in Northern Uganda is significant. Enhanced screening and early identification of dementia is recommended in this setting.

## Background

Dementia is the seventh leading cause of death, a major cause of disability, dependency among older people worldwide, and more than 55 million people live with dementia, with an estimated 10 million new cases annually^1^. Whilst many cases have been recorded in the high-income countries, it is already known that [ODL1] two thirds of people living with dementia (PLWD) reside in low and middle-income countries (LMICs) with very limited access to social protection, services, support and care^2^. Current evidence suggests that up to 90% of people with dementia in LMICs do not receive a dementia diagnosis^3^.

Uganda has an increasingly aging population, contributing to nearly 5% of the total population and it is expected to increase to 5.5 million by 2050^4^. Currently the life expectancy of Ugandans has increased to 63 years, and despite this demographic transition, dementia is not yet a national priority^5^.

The prevalence of dementia varies widely in Africa from 2.3% to 20.0%^6^. Therefore, validated culture-sensitive cognitive tools not influenced by educational levels and language differences are critically needed for proper dementia screening and diagnosis across Africa^6^. Several variables have been reported as potential risk and protective factors for dementia, such as vascular disease, lifestyle, psychosocial and psychological factors, infectious diseases, genetic factors, and carbon monoxide poisoning which generally is grouped under modifiable and non-modifiable factors^7^.

Few studies of the prevalence and correlates of dementia in sub-Saharan Africa have been conducted to guide clinical care. Therefore, in this study we determined the prevalence and factors associated with dementia among older persons in Northern Uganda.

## Methods

### Study design

We conducted a cross-sectional, out-patient based hospital study from March to May 2022

### Study procedure

We used the outpatient register for a specific day as the sampling frame, and we then randomly selected the first patient who was 60 years and above then used systematic sampling until the desired sample size was achieved. If the patient did not meet the inclusion criteria, then the next patient above 60 years was considered for enrolment. Informed consent was sought from [ODL2]all the study participants or their legal representatives before enrolment into the study. Participants were required to have an immediate caregiver to respond to any questions related to their activities of daily living. We excluded participants who were deaf and blind and those who didn't have immediate caregivers to respond to the study questions and undertake cognitive tests.

### IDEA

We used the IDEA cognitive screen which is a 6-item brief dementia screening instrument validated in LMICs. The items 1–4 is taken from the Community Screening instrument for dementia. These involve questions that tap into being able to name a bridge from a description of its use, knowing the day of the week, knowing the name of the village chief/town mayor/city governor and naming as many animals as possible in one minute (score 2 for ≥ 8 animals, score 1 for 4–7 animals, score 0 for 0–3 animals). Item 5 is taken from Consortium to Establish a Registry for Alzheimer's Disease (CERAD) 10-word recall test, with recall of 10 common words after 5 minutes delay (score 1 point for each word up to a maximum of 5 points). The sixth item is designed to measure praxis and involves a matchstick design test, with scores ranging from 0 (no matchsticks placed correctly), to 3 (all four matchsticks placed correctly in the shape of a rake). The maximum possible score was 15 and the minimum 0, with a higher score indicating better cognitive function (8). The screen was administered in Luo language. Patients who scored 8-9 were be termed probable dementia, while those with dementia were defined as those that screened a cognitive score ≤ 7. Those with probable dementia was again subjected to IDEA-IADL study questionnaire.

### IDEA-IADL

The IDEA-IADL is an 11-item informant-based scale assessing IADLs was administered by trained research assistants to caregivers or relevant informants of patients who scored 8-9 on the IDEA cognitive screen at the time of interview. Each item had a four-point scale (0–3) from zero (‘cannot do this’) to three (‘can do it with no problems, do not need help’) with a total score of 33. A cut-off of ≤ 23 was considered indicative^8^.

### Factors of association questionnaires

Following cognitive assessment for dementia, we then administered a pretested study questionnaire to patients to elicit other variables which included: vascular disease, lifestyle behaviors, psychosocial and psychological factors, infectious disease exposure, and family history of dementia.

Each variable was enumerated as being either present (yes) or absent (no). Where necessary, the patients' caretaker gave further clarification during the interview. Study participants ages were crosschecked with their national identification cards. Formal education was defined as the number of years spent in school: none, 1–7 years (primary school), 8–11 years (ordinary level school), and 12 or more years (advanced level and tertiary education). Family history of dementia was elicited with a single self-report item identifying a first-degree relative (sibling or parents) who has ever showed signs of dementia or received a diagnosis of dementia.

Life time history of cigarette smoking and alcohol consumption were elicited using self-report. High-fat dietary intake assessed with a single item which elicited a lifetime history of high-fat intake, defined as regular, frequent consumption of animal products (e.g., milk, meat, ghee) or ground nuts during the ages of 25-45 years. Religious integration was defined as participating in religious activities in addition to community prayer.

To assess physical activity prior to 60 years of age, we asked a single question about heavy manual labor, riding a bicycle at least three days a week, or engaging in 30 minutes of exercise at least three times a week.

Lifetime history of traumatic head injury was defined as ever experiencing head injury accompanied by loss of consciousness. We also elicited lifetime history of syphilis, tuberculosis, human immunodeficiency virus, bacterial meningitis, cerebral malaria, type II diabetes mellitus, cerebrovascular accident, hypertension, epilepsy. Study participants' responses were crosschecked against any accompanying medical records to the hospital. All study tools were interviewer-administered in the local language (Luo). Survey questions were written in English, translated from English into Luo, and then back translated to verify fidelity to the original wording.

### Data analysis

Cleaned data was entered into an Excel sheet Windows 10. Double entry was done followed by a comparison of data sets with hard copies to find discrepancies. The data set was then exported to STATA version 16.0 for analysis. The IDEA-IADL provided an estimate of the screening prevalence of dementia. Data were summarized as frequencies (percentages) that is prevalence of Dementia. We then compared the proportions of those with probable dementia across different subgroups. Bivariate analysis of associated factors using cross tabulation; categorical data were analyzed using the chi square test. A p≤0.05 was statistically significant. Multivariate analysis was done for those variables that had a p value of 0.2 on bivariate analysis, using forward multivariable logistic regression model and this was reported in adjusted odds ratio and p values.

### Ethics approval and consent to participate

Ethical approval to conduct this study was obtained from the School of Medicine Research and Ethics Committee (SOMREC) registration number Mak-SOMREC-2021-93, and administrative clearance from St. Mary's Hospital Lacor, Gulu. Written informed consent was obtained from the participants or their caretakers before enrolment into the study. Information got was treated with at most confidentiality, used for study purposes and clinical management only. In addition, instead of names, participant identification numbers were indicated on the data collection tools. All participants who screened positive dementia had their relevant information shared with the attending doctor to facilitate further clinical assessment and management.

## Results

### Characteristics of the study sample

Of the 271 patients that were recruited into the study; nearly 61% were males (165/271) with a median age of 68 years. About fifty-six-point five percent were within the age group 60 to 6y years. Most of the participants had less 7 years of formal education and most of them were peasant farmers 186 (68.6%). In addition, more than two thirds of the patients were Catholics 182 (67.2%) and majority of them were married 195 (72%), Table 1.

**Table 1:** Characteristics of the study population

Variable		
Age, median, 68 (IQR, 63-74), years	Frequency	Percentage
60-69	153	56.5
70-79	83	30.6
80-89	32	11.8
>=90	3	1.1
**Sex**		
Male	165	60.9
Female	106	39.1
**Occupation**		
Peasant	186	68.6
Retired	31	11.4
Business	30	11.1
Civil servant	15	5.5
Self employed	9	3.3
**Marital status**		
Married	195	72
Divorced	13	4.8
Never married	3	1.1
Widowed	60	22.1
**Education**		
Some, but did not complete primary	99	36.5
Completed primary	74	27.3
Completed secondary	32	11.8
Completed tertiary	30	11.1
None	36	13.3
**Religion**		
Catholic	182	67.2
Anglican	66	24.4
Muslim	7	2.6
Other	16	5.9

### Prevalence of dementia

**Figure 1:**
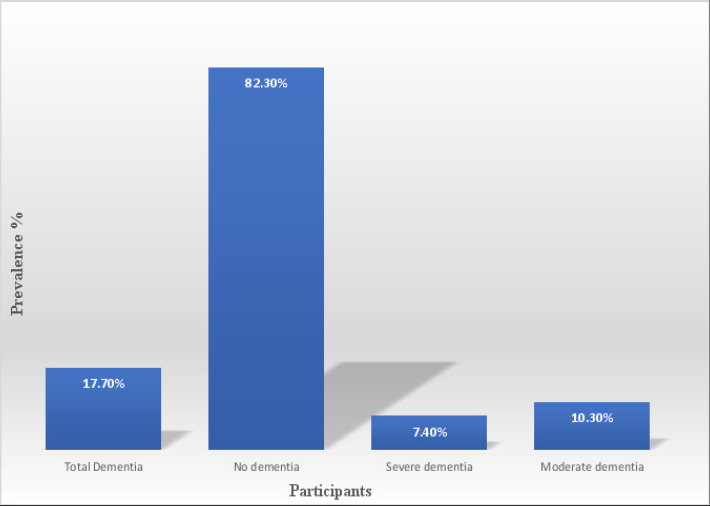
Prevalence of dementia

Among the 271 study participants, 17.7% (48) participants met the criteria of cognitive impairment (Moderate and severe dementia). Of these females accounted 48.3% (21/48) while males accounted for 51.7% (27/48). Severe dementia accounted for 7.4% (20/271) participants with score of ≤7 on the IDEA cognitive screen meanwhile moderate dementia accounted for 10.3% (28/271) participants. This was measured by a score of 8-9 on the IDEA cognitive screen and a subsequent score of ≤23 on the IDEA-IADL. Dementia was present at 10.4%, among age groups of 60-69years, and 16.9% among age groups 70-79 years. Lastly majority of dementia more than one half of patients 80 years and above were found to be having dementia and again a higher proportion of dementia occurred in females. In conclusion the prevalence of dementia in this study was found to be 17.7% which combined moderate and severe categories, Figure 1.

### Factors associated with dementia

On bivariate; analysis, there were some factors that had significant association with development of dementia. Socio-demographic factors; age 80-89 years 35% (n=17), family history of dementia 31.3%, (n=17) and behavioral factors: physical activity 56.1% (n=125) and cognitive exercise 33.2% (n=74). Other factors with some positive associations with dementia were: marital status, history of depression 58.3% (n=28), and two vascular factors which included history of heart failure 12.5% (n=6) and stroke 10.4% (n=5), Table 2.

**Table 2:** Bivariate analysis of factors of association

Variable	All (n=271) Freq (%)	Dementia		P value
		Yes (n=48) Freq (%)	No (n=223) Freq (%)	
**Age, years**				
**60-69**	153(56.5)	16(33.3)	137(61.4)	<0.001
**70-79**	83(30.6)	14(29.2)	69(30.9)	
**80-89**	32(11.8)	17(35.4)	15(6.7)	
**>=90**	3(1.1)	1(2.1)	2(0.9)	
**Marital status**				
**Divorced**	13 (4.8)	2(4.2)	11(4.9)	0.018
**Married**	195(72)	27(56.3)	168(75.3)	
**Never married**	3(1.1)	0(0)	3(1.4)	
**Widowed**	60(22.1)	19(39.6)	41(18.4)	
**Family history of dementia**				
No	230(84.9)	33(68.8)	197(88.3)	0.001
Yes	41(15.1)	15(31.3)	26(11.7)	
Depression	105(38.7)	28(58.3)	77(34.5)	0.003
Heart failure	16(5.9)	6(12.5)	10(4.5)	0.044
Stroke	11(4.1)	5(10.4)	6(2.7)	0.028
Physical activity	130(48)	5(10.4)	125(56.1)	<0.001
Cognitive exercise	78(28.8)	4(8.3)	74(33.2)	<0.001

When the patient's covariates were entered simultaneously into a multivariable logistic regression model, the following factors retained a statistically significant association with dementia: Participants aged 80-89 years had an almost 10-fold higher odds of having dementia, aOR; 9.7, 95CI: 4.08 — 23.07, p< 0.001. Likewise, patients who reported having family history of dementia were almost 3.5 folds higher odds of having dementia, aOR; 3.4, 95% CI, 1.62-7.04, p= 0.001. Additionally, among the 230 participants without a family history of dementia, 33 (69%) were found to have developed dementia. This suggests that other factors, beyond family history, play a significant role in the development of dementia. Furthermore, participants who report history of depression were also 3 folds more likely to have dementia at multivariate analysis, aOR; 2.7, 95CI, 1.40-5.02, p= 0.003. Meanwhile modifiable factor, physical activity was a highly associated protective factor aOR; 0.1, 95CI, 0.03-0.24, p< 0.001. Lastly, involvement in cognitive exercise was also an associated protective factor, aOR; 0.2, 95CI, 0.06-0.53, p<0.002, Table 3.

**Table 3:** Multivariate analysis of factors of association

Variable	Adjusted Odds ratio	95% CI	P value
**Age, years**			
**60-69**	1.0		
**70-79**	1.7	0.80-3.77	0.162
**80-89**	9.7	4.08-23.07	<0.001
**>=90**	4.3	0.37-49.89	0.246
**Marital status**			
**Divorced**	1.0		
**Married**	0.9	0.19-4.26	0.889
**Never married**	N/A		
**Widowed**	2.5	0.51-12.65	0.252
**Family history of dementia**			
**No**	1.0		
**Yes**	3.4	1.62-7.04	0.001
**Depression**			
**No**	1.0		
**Yes**	2.7	1.40-5.02	0.003
**Physical activity**			
**No**	1.0		
**Yes**	0.1	0.03-0.24	<0.001
**Cognitive exercise**			
**No**	1.0		
**Yes**	0.2	0.06-0.53	0.002

## Discussion

In this hospital-based study of 271 older age adults at St. Mary's Hospital Lacor Outpatient Department, we estimated the prevalence of dementia to be 17.7%. This estimated prevalence is closer in similarity to the 20% estimated prevalence rate reported in a recently published community-based survey from rural South western Uganda, which had a similar age distribution of study participants but used a different screening instrument (i.e., the brief CSID)^9^. Notably, our study categorized patients with positive dementia screens as Severe dementia with proportion of 7.4% and Moderate Dementia at 10.3%. Similarly, Stella-Maria Paddick et al. reported a higher prevalence of 21.6% using the 10/66 diagnostic criteria following their study in Tanzania, Hai District^10^, with similar demographic profile in comparison to our study. In addition, another hospital-based cross-sectional study in non-psychiatric wards in Uganda by Nakasujja et al. reported a prevalence of 14% and again more than one half of these were 80 years and above^11^. However, the prevalence findings in this study are higher than reported in regions of sub-Saharan Africa 4.76% from a report of key messages on the prevalence of dementia by World Health Organisation^12^. Again, lower estimates than in this study was reported in the Swiss study which assessed dementia in specialized residential homes of people with disability and found a prevalence of 5.8%^13^. In another study of prevalence of dementia in rural Kilimanjaro, the age-adjusted prevalence of dementia was 4.6% in those aged ≥60 years and 8.9 in those aged ≥70 years^14^. However, there is a wide variation in prevalence of dementia in Africa, ranging from as low as 2.3% to as high as 20%^6^ which generally encompasses the figure reported this study.

Several factors influence the markedly varied prevalence of dementia reported in SSA in many studies including this one. Firstly, this is a vast region with a population of about 1.3 billion and peak increase in population is projected to occur by 2052 with East Africa in particular forming the single largest population block^15^. These increases in population coupled with marked differences in percentages of older people in this region and several ethnic groups, with different cultures, languages, diets, and traditions could account for the significant variations of dementia prevalence reported^15^,^16^. Furthermore, in this poor but rapidly developing region of the world where people are living longer, and having fewer children, dementia becomes much more evident and pronounced due to longevity^17^. Again, numerous dementia screening tools have been developed and used in Africa which is majorly low-income region with associated high illiteracy rates, and some of these tools used in high income countries were probably incorrectly validated for use in low-income setting^18^. The variations in screening tools used therefore most likely reflects the marked variation in prevalence reported across regions.

Our study identified several independent factors of association of dementia; two non- modifiable factors age and family history of dementia. In addition, depression a modifiable factor was also significantly associated with dementia meanwhile of note two protective factors for the development of dementia found were, history of physical activity and engagement in cognitive exercises.

As generally expected, advancing age had a statistically significant positive correlation with dementia, similar to what has been demonstrated in other studies^7^,^9^,^17^,^19^-^22^. The study finding add more weight to the statement from WHO that age is the strongest known risk factor for dementia however it is not an inevitable consequence of biological ageing^23^. Notably, consistent with prior work, this study found that family history of dementia was associated with dementia and this finding is similar to a large cross-sectional study in Netherland which concluded that middle age individuals with past family history of dementia were at increased risk of development of dementia^24^. Similarly, Robert et. al, found that people with a first-degree family hisory of Alzheimer's disease are at an increased risk of developing dementia possibly due to hippocampal cortical thinning^25^. In another population-based study cohort study by Wolters et. al, parental history was associated with risk of dementia independently of known genetic risk factors^26^.

This study also identified physical activity as an associated protective factor and is similar to recommendation by the Lancet Commission which entails: interventions for risk factors including exercise to potentially delay or prevent a third of dementia cases^7^. In addition, prior work has shown that physical activity is a promising intervention for the prevention and non-pharmacological treatment of dementia^27^. Similarly prior work which evaluated the effect of physical activity on dementia reported a reduced risk of total dementia^28^-^30^ consistent with our finding.

History of depression which we found was significantly associated with dementia is supported by several studies including the 2020 dementia report of the Lancet commission which outlined 12 modifiable risk factors of dementia^7^. This study was carried out in northern Uganda a region which was affected by war for over 20 years and many studies have pointed out high prevalence of depression^31^. Whether the 20 years of war in northern Uganda has contributed to this finding in relation to dementia is not yet known. However, several prior works have alluded to the important association of depression with dementia^32^-^35^. People in Northern Uganda and especially our study population might have been exposed to depression as a result of war.

Furthermore, this study also found cognitive exercises a preventive associated factor again consistent with recommendation from the Lancet commission on interventions to prevent dementia by strengthening the functioning and plasticity of neural circuits^7^. Prior works have alluded to the important and significant reduction in total dementia risk in association with cognitive exercises^28^,^36^,^37^.

### Study limitations

Interpretation of our findings is subject to several limitations; firstly, this was a single center study which conducted in Northern Uganda and thus limited in representing the various ethnics groups and regions in Uganda since the prevalence of dementia and factors of association will vary from region to region and ethnicity. Secondly due to limited resources, this study didn't obtain clinical diagnosis of dementia using structured clinical interviews and might have overestimated the prevalence of dementia. However, the IDEA-IADL a dementia screening tool validated for use in LMICs has shown strong evidence of validity in screening for dementia. Lastly, early and midlife exposure variables were measured with self-report. Cognitive impairment in patients could have caused them to recall these variables with error.

With these limitations in mind, and putting forward the importance of understanding the burden of dementia and associated factor in resource limited northern Uganda in order to establish a care pathway through early detection with simple and accurate brief cognitive assessment tool like IDEA-IADL. This study is therefore crucial to inform policy recommendations for diagnosis of dementia establishment of preventive interventions at local levels and also a foundation base from which future research in genetics and other aspects of dementia can be relied on.

## Conclusions

In this outpatient hospital-based study sample of older adult patients, almost one-fifth screened positive for dementia which was similar to another community-based study in rural western Uganda, however higher prevalence than generally reported in studies elsewhere in Sub-Saharan Africa. The discrepancy could have resulted from better case finding by the screening tool or regional differences in the prevalence of dementia and lastly different cognitive tools used within Africa. Further studies are recommended to look at regional differences in the prevalence of dementia using similar culturally accepted tool like IDEA-IADL.

## Data Availability

The full dataset generated and analyzed during the current study are not publicly available in order to maintain the privacy of the individuals interviewed during this study. De-identified data can be made available by the corresponding author on reasonable request.

## Abbreviations

IDEA-IADL: Identification of Dementia in Africa Instrumental Activity of daily living
AOR: Adjusted odds ratio
CI: Confidence interval
IDEA: Identification of Dementia in Africa
IQR: Interquartile range
